# The Regulation of Peripheral Metabolism by Gut-Derived Hormones

**DOI:** 10.3389/fendo.2018.00754

**Published:** 2019-01-04

**Authors:** Emily W. L. Sun, Alyce M. Martin, Richard L. Young, Damien J. Keating

**Affiliations:** ^1^College of Medicine and Public Health, Flinders University, Adelaide, SA, Australia; ^2^Nutrition and Metabolism, South Australian Health and Medical Research Institute, Adelaide, SA, Australia; ^3^Adelaide Medical School, The University of Adelaide, Adelaide, SA, Australia

**Keywords:** GLP-1, PYY, serotonin, GIP-glucose-dependent insulinotropic peptide, oxyntomodulin, ghrelin, enteroendocine cells, insulin-like peptide 5 (INSL5)

## Abstract

Enteroendocrine cells lining the gut epithelium constitute the largest endocrine organ in the body and secrete over 20 different hormones in response to cues from ingested foods and changes in nutritional status. Not only do these hormones convey signals from the gut to the brain via the gut-brain axis, they also act directly on metabolically important peripheral targets in a highly concerted fashion to maintain energy balance and glucose homeostasis. Gut-derived hormones released during fasting tend to be orexigenic and have hyperglycaemic potential. Conversely, gut hormones secreted postprandially generally promote satiety and facilitate glucose clearance. Although some of the metabolic benefits conferred by bariatric surgeries have been ascribed to changes in the secretory profiles of various gut hormones, the therapeutic potential of the enteroendocrine system as a viable target against metabolic diseases remain largely underexploited, except for incretin-mimetics. This review provides a brief overview of the physiological importance and highlights the therapeutic potential of the following gut hormones: serotonin, glucose-dependent insulinotropic peptide, glucagon-like peptide 1, oxyntomodulin, peptide YY, insulin-like peptide 5, and ghrelin.

## Introduction

Gut enteroendocrine cells (EECs) are specialized secretory cells that are sparsely scattered throughout the mucosal epithelium of the gastrointestinal (GI) tract and which constitute the largest endocrine organ by mass in the body (1). EECs have the capacity to “sample” luminal contents on the apical membrane, and collectively release more than 20 different hormones basolaterally in response to a variety of stimuli. With each having their own specialized functions, EECs have been historically characterized by their hormonal profiles, such as glucagon-like peptide 1 (GLP-1)- and peptide YY (PYY)-secreting L-cells and serotonin (5-hydroxytryptamine, 5-HT)-secreting enterochromaffin (EC) cells. It is now accepted that there are vast overlaps in the secretory profiles of EECs (2) and the “one cell type, one hormone” dogma is widely rejected. Studies using transgenic mice expressing fluorescent reporter proteins driven by promoters of different gut hormones revealed that multiple hormones can be simultaneously expressed by an individual EEC (3, 4) while high-resolution microscopy shows that these different hormones are packaged into separate vesicles within the EEC (5–7). Expression of EEC hormones are also regionally distinct, as many gut hormones are confined to specific regions of the gut, while a subset, such as 5-HT and somatostatin, are present throughout the GI tract (8, 9). Enteroendocrine hormones are implicated in a wide range of physiological functions including gastrointestinal motility, appetite control, and glucose homeostasis (10). Mounting evidence demonstrates the importance of gut hormones in regulating peripheral metabolism in health and disease and as a result, a myriad of therapeutics against metabolic diseases that are based on the actions of specific gut hormones are currently under clinical development (11–13). As such, it is timely to review the literature regarding the metabolic actions of these gut hormones: serotonin, glucose-dependent insulinotropic peptide, glucagon-like peptide-1 (GLP-1), oxyntomodulin, peptide YY (PYY) and ghrelin. We also discuss the metabolic actions of insulin-like peptide 5, a recently characterized gut hormone that are co-secreted with GLP-1 and PYY.

## Serotonin

Serotonin (5-HT) is produced by enterochromaffin (EC) cells, which constitute ~50% of the total EEC population and are scattered throughout the length of the gut, from the stomach to the distal colon (2, 8). Although better known for its actions in the CNS, more than 90% of total body 5-HT is synthesized by EC cells, the majority of this being stored in platelets (14, 15). Tryptophan hydroxylase 1 (TPH1) is the rate-limiting enzyme of 5-HT synthesis in specific non-neuronal cells and its expression in the gut mucosa is limited to EC cells. EC cells have the capacity to sense a wide range of stimuli present in the gut lumen such as glucose and fructose (16, 17), the medium chain fatty acid, lauric acid (18), various tastants and olfactants (19), and to secrete 5-HT in response. 5-HT secretion from EC cells is also regulated by mechanical stimuli (20), and neural and endocrine input such as adrenergic stimulation and GABA and somatostatin inhibition (21). In addition, microbial metabolite signals from the gut microbiome also augment colonic EC cell density, 5-HT secretion and circulating 5-HT levels (22).

Although traditionally regarded as a regulator for gastric motility (23–25) and more recently, a mediator in the pathogenesis of inflammatory intestinal disorders (14, 26), mounting evidence highlights gut-derived 5-HT as a modulator of peripheral metabolism (27, 28). Under fasting conditions, gut-derived 5-HT, together with glucagon, markedly increases hepatic glucose output, a main driver of fasting euglycaemia, by increasing hepatic gluconeogenesis and glycogenolysis (29), while inhibiting glucose uptake and glycogen synthesis in the liver (30). In conjunction, 5-HT promotes lipolysis within white adipocytes to liberate free fatty acids (FFAs) and glycerol (30) as key substrates for hepatic gluconeogenesis, and further enhance hepatic glucose output. Moreover, gut-derived 5-HT promotes energy conservation and weight gain by reducing energy expenditure, via actions to attenuate thermogenesis in brown adipose tissue (31) and inhibit the browning of white adipose tissue (32).

Gut-derived 5-HT also attenuates the release of several metabolically important blood glucose-lowering chemokines, such as adiponectin from adipose tissue (33), and bone-derived osteocalcin and lipocalin 2 (34–36), through inhibition of osteoblast proliferation (37). Significantly elevated mucosal *TPH1* expression in obese humans (38, 39) and elevated levels of circulating 5-HT in individuals with type 2 diabetes (T2D) (40–42) or obesity (38) has been reported. Inhibition of intestinal TPH1 in mice, through tissue-specific ablation or pharmacological inhibition, conveys protection from high-fat diet (HFD)-induced dyslipidaemia and glucose intolerance (30–32). This confirms a causative role of elevated gut-derived 5-HT as a driver of metabolic dysfunction. TPH1 inhibition also protects mice from diet-induced obesity (DIO) (31). However, despite clear evidence that EC cell-derived 5-HT negatively impacts energy balance and glucose homeostasis, the underlying causes of elevated 5-HT levels with obesity and T2D remain unclear. Likely drivers of increased circulating 5-HT are increased density or glucose-sensitivity of duodenal EC cells, as evidenced in obese human duodenal EC cells (38), however molecular mechanisms underlying this are not understood. Due to the heterogeneity in 5-HT receptors across many tissues (43), targeting 5-HT receptor signaling pathways may not be a viable therapeutic target for treatment of metabolic disease.

## Glucose-dependent Insulinotropic Peptide

Glucose-dependent Insulinotropic Peptide (GIP) is a 42-amino acid peptide hormone produced by K cells located primarily in the proximal small intestine (44). GIP is secreted in response to nutrient stimulation and exerts its actions by binding to the GIP receptor (GIPR) expressed by pancreatic islet cells (45), adipocytes (46), bone cells (47), and the CNS (48). Circulating GIP is rapidly degraded by dipeptidyl peptidase IV (DPP4), a serine protease that is widely expressed throughout the body, especially in endothelial cells (49). The insulinotropic effect of GIP, together with GLP-1, accounts for more than 70% of postprandial insulin secretion (50). GIP also increases insulin biosynthesis (49), promotes β-cell proliferation and inhibits β-cell apoptosis (51). The insulinotropic effects of GIP are dramatically attenuated in T2D patients (52, 53), and this is believed to be a major contributing factor to impaired postprandial insulin secretion in these individuals. Moreover, the insulinotropic potency of GIP is markedly reduced in non-diabetic, first-degree relatives of T2D patients (54), suggesting altered GIP signaling could be one of the many predisposing factors for T2D later in life. While the mechanism underlying the diminished insulin response to GIP in T2D has not yet been fully elucidated, receptor downregulation (55) and desensitization (56) have been suggested as potential causes. Although GIP only stimulates glucagon secretion under hypo- and euglycaemic conditions in healthy individuals (57), its glucagonotropic effect is exaggerated in T2D patients during hyperglycaemia (58). This further worsens glycaemic control in these patients, and in combination with the reduced insulinotropic potency renders GIP an undesirable therapeutic target for T2D treatment.

The anabolic properties of GIP closely resemble those of insulin, as it promotes lipid uptake and inhibits lipolysis in adipocytes (59). Several studies have reported elevated GIP levels in obese humans (60, 61). Elevated GIP levels and duodenal K cell hyperplasia (62) have also been reported in HFD-treated mice, while *Gipr* deficiency protects mice from HFD-, leptin deficiency- or ovariectomy-induced weight gain (63, 64). GIP also induces osteopontin expression in adipocytes (65), an adipokine associated with obesity-related systemic low grade inflammation (66, 67). Adipocyte-specific *Gipr* ablation protects mice from HFD-induced insulin resistance and hepatic steatosis, potentially by reducing circulating levels of pro-inflammatory cytokines (68). However, the obesogenic effects of GIP are only apparent during nutrient excess, as chow-fed *Gipr* and *Gip* knockout animals are of similar weight as their wild type counterparts (69). The role of GIP in energy balance is further complicated by paradoxical findings that mice overexpressing *Gip* were leaner than wild type controls, when fed either a standard-chow or HFD (70). Such observation could be attributed to the anti-apoptotic effect of GIP on osteoblasts (71), as osteoblast-derived hormones such as osteocalcin and lipocalcin 2 are implicated in regulating peripheral metabolism and modulate food intake (36, 72). Furthermore, powerful evidence has emerged to show that GIPR signaling can enhance GLP-1-induced weight loss (11, 73).

## Glucagon-like Peptide 1

Glucagon-like Peptide 1 (GLP-1) is an incretin hormone secreted by enteroendocrine L cells upon ingestion of nutrients, including glucose (74), and typically within 10–15 min into the postprandial period (75). GLP-1 is subjected to rapid degradation by DPP4 (76) and acts via the GLP-1 receptor (GLP-1R) expressed on a myriad of target tissues (75). GLP-1 plays a key role in maintaining glucose homeostasis, as it markedly increases glucose-stimulated insulin secretion (GSIS) (77) and attenuates hepatic glucose production, independent of its effect on pancreatic islets (78, 79). There is growing appreciation that a considerable portion of the glucose-lowering effect of GLP-1 is underscored by its inhibitory effect on gastric motility (80–83) and its glucagonostatic action (84, 85), which are preserved in obese and T2D patients (86, 87). Unlike GIP, the potent insulinotropic effect of GLP-1 is predominantly preserved in T2D patients and, thus, has led to the development of GLP-1-based therapies for preserving blood glucose control in individuals with T2D.

**Figure 1:**
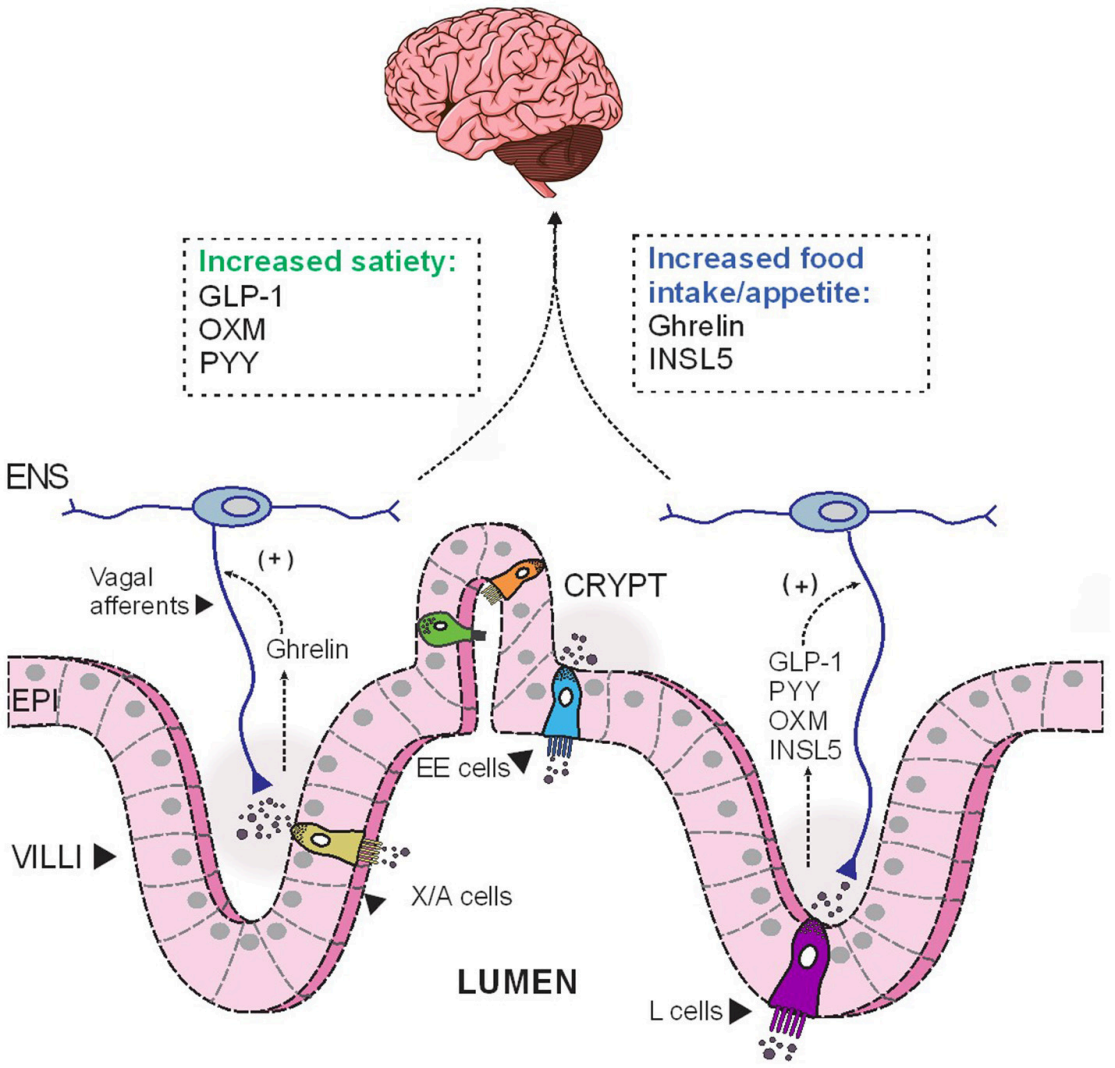
The opposing effects of anorectic and orexigenic gut hormones on food intake. Depending on the luminal stimulation, EE cells release different hormones basolaterally, which then diffuse across the lamina propria to act on their corresponding receptors expressed on nerves endings of vagal and enteric afferents. These hormonal cues are subsequently relayed to the CNS to modulate food intake. EE, enteroendocrine; ENS, enteric nervous system; EPI, epithelial cells; INSL5, insulin-like peptide 5; GLP-1, glucagon-like peptide 1; OXM, oxyntomodulin; PYY, peptide YY.

In addition to its multifaceted glucose-lowering effect, GLP-1 regulates energy balance and adiposity through its effects on satiety and appetite. The acute anorectic effect of GLP-1 is mediated by GLP-1R located on vagal afferents (88), which relays the signal to appetite control centers, namely the NTS in the brainstem, to reduce food intake (89) (Figure 1). GLP-1R are also widely expressed in brainstem and hypothalamic regions implicated in appetite control (90). In humans, acute administration of pharmacological doses of GLP-1 significantly induce satiety and reduce food intake (91–93). Furthermore, exaggerated postprandial GLP-1 response is believed to contribute to the increased satiety reported by many gastric-bypass surgery patients (94–96). However, a recent clinical study reported that the infusion of exendin 9-39, a GLP-1R antagonist, did not affect *ad libitum* food intake in post-RYGB patients, although the authors also reported a concomitant increase in plasma levels of the anorexigenic hormone PYY (discussed below), which might offset the orexigenic effect of GLP-1R antagonism (94). The DPP4-resistant GLP-1R agonist, liraglutide, is now in clinical use as a weight-loss therapeutic in obese/overweight individuals (97). GLP-1 is also implicated in regulating hedonic eating through GLP-1Rs located elsewhere in the brainstem (98–100). Peripherally administered GLP-1R agonists may also act directly on GLP-1R at other sites in the brain, notably circumventricular organs and some hypothalamic regions with fenestrated capillaries (101–103). Indeed, Liraglutide can directly activate anorectic POMC/CART neurons in rodents and thus, indirectly inhibit orexigenic AgRP/NPY neurons in the arcuate nucleus (ARC) to reduce food intake (101). As endogenous GLP-1 has a very short half-life, these central actions are likely to be more relevant during therapeutic use of DPP4-resistant GLP-1R analogs, or in post-gastric bypass surgeries, in which GLP-1 “equivalent” levels, or postprandial GLP-1, respectively, are augmented and sufficient to elicit anorectic responses at these CNS targets.

## Oxyntomodulin

Oxyntomodulin (OXM) is a 37-amino acid peptide that contains the entire amino acid sequence of glucagon (104) and is co-secreted with GLP-1 by enteroendocrine L cells at an equimolar ratio (105). Although an endogenous OXM receptor has not been identified, OXM exerts weak agonist activity on GLP-1R (106) and the glucagon receptor (GCGR) (107). Nevertheless, pharmacological levels of OXM (sufficient to activate GLP-1R and GCGR) have shown anti-obesity effects in humans, by significantly reducing appetite (108, 109) and increasing energy expenditure (110). In addition, OXM treatment improved glucose tolerance in high-fat fed mice by potentiating GSIS (111), in a glucose-dependent manner (112), and has an anti-apoptotic effect on β cells (112). OXM infusion significantly reduced glycaemic excursion by augmenting GSIS in obese subjects with or without T2D (113). Such observations prompted the investigation into the potential metabolic benefits of GLP-1R and GCGR co-activation (114, 115), which led to the subsequent development of GLP-1R/GCGR co-agonists (73, 116) and, later, GIPR/GLP-1R/GCGR tri-agonists (117). These agonists have shown impressive anti-obesity effects in preclinical models and are currently being evaluated in phase 2 clinical trials (118).

## Peptide YY

Peptide YY (PYY) is co-localized with GLP-1 in enteroendocrine L cells (7, 119) and is co-released with GLP-1 postprandially, in proportion to caloric intake (119, 120). In contrast to GLP-1, which is present in sufficient amount in the duodenum to account for the immediate postprandial surge, PYY abundance is very low in the upper gut and increases distally from the ileum toward to colon (121, 122). Thus, postprandial PYY release under normal physiological conditions is likely to be mediated through paracrine and neural mechanisms (123). An exaggerated postprandial PYY response is observed in gastric bypass patients, and is likely attributed to the increased flow of nutrients into the PYY-rich distal gut, which can directly stimulate L cells (124, 125). Human PYY circulates in two active forms: PYY_1−36_ and PYY_3−36_, the latter being an active cleavage product of the former by DPP4 (126). Both are key mediators of the “ileal brake,” a local feedback mechanism triggered by the arrival of nutrients in the ileum that inhibits gastric and pancreatic secretions and proximal intestinal motility (127). The physiological effects of PYY are mediated through a family of NPY receptors (termed Y1, Y2, Y3, Y4, and Y5), which are differentially expressed in a wide range of tissue including enterocytes, myenteric and submucosal neurons and extrinsic primary afferent nerve fibers (123).

Exogenous PYY administration significantly reduces food intake in both obese and lean subjects (128, 129). *Pyy-*deficient mice are hyperphagic and obese (130) while *Pyy* overexpression protects mice against obesity induced by HFDs or leptin deficiency (131). Although the “ileal brake” mechanism contributes to its satiating effect (132), PYY_3−36_ induces satiety primarily by targeting the hypothalamus. The role of PYY as a satiety hormone has been debated, as several independent research groups did not reproduce the anorectic effect in humans reported in the original study by Batterham et al. (133). Moreover, due to its nauseating effect at higher doses (134–136), PYY has not been pursued as an anti-obesity target.

PYY infusion in humans had limited effects on plasma glucose, insulin or glucagon levels on its own (128, 137), nor did it affect glucose excursion and insulin levels during intravenous (138) or oral glucose challenge (136). PYY has trophic effects on pancreatic β cells (139), but such effects are believed to be mediated by islet-derived, rather than gut-derived PYY (140). However, as postprandial PYY levels after gastric bypass surgeries are elevated several folds, it may be possible for gut-derived PYY to exert protective effect on β cells in these settings.

## Ghrelin

Ghrelin is an orexigenic hormone secreted by X/A cells present in the mucosa throughout the length of the GI tract, with the highest abundance in the gastric fundus. Circulating ghrelin is significantly elevated during fasting and attenuated upon meal initiation. Post-translational acylation of the ghrelin peptide by ghrelin O-acyl-transferase (GOAT) is crucial for its activity at its endogenous receptor, growth hormone (GH) secretagogue receptor (GHSR1a) (13). GHSR1a is highly expressed in the CNS and is capable of stimulating GH release from the anterior pituitary (13), and lower levels of expression are found in the periphery including the small intestine and pancreatic islets (141). Exogenous ghrelin reliably increases food intake in various species, including humans (142). The orexigenic action of ghrelin is mediated through direct stimulation of the orexigenic AgRP/NPY neurons and concomitant inhibition of the anorectic POMC/CART neurons in the ARC (143, 144). Weight loss achieved through caloric restriction is accompanied by marked elevation in circulating ghrelin (145), which increases feeding drive and has therefore been ascribed as a natural defense against weight loss. Ghrelin is also an anabolic hormone that drives lipogenesis, independent of its effect on appetite (146). Altogether, the orexigenic and anabolic properties of ghrelin renders the ghrelin-GOAT-GHSR1a axis an attractive anti-obesity target. Pharmacological blockade of GOAT or GHSR1a have yielded promising results in preclinical models of obesity (147–150). However, genetic disruption of different components of the ghrelin-GOAT-GHSR1a axis in mice did not have the anticipated anorectic or anti-obesity effects (151–154). Neither *Ghrelin* nor *GOAT* deficiency rescue the obese and hyperphagic phenotype of *ob/ob* mice (152, 155). As such, these data indicate a dispensable role for ghrelin in the regulation of feeding and bodyweight, and that the role of ghrelin in increasing feeding drive may be limited to fasting conditions.

Contrary to its limited role in feeding behavior, ghrelin is a key regulator of glucose homeostasis. Exogenous ghrelin markedly increases blood glucose levels in humans, while genetic ablation of ghrelin or its receptor improve glucose tolerance in HFD-fed and *ob/ob* mice (152, 156). Ghrelin receptor signaling, specifically in hypothalamic AgRP/NPY neurons, is a critical countermeasure to prevent hypoglycaemia (143). Mice with attenuated ghrelin signaling, due to *GOAT*-deficiency or ghrelin cell ablation, have a blunted counter-regulatory GH response, and display profound fasting-induced hypoglycaemia (157, 158). Ghrelin protects against hypoglycaemia by triggering the direct release of GH from the anterior pituitary (159), increasing glucagon secretion (160) and inhibiting insulin secretion (161, 162). Ghrelin can protect mice from hypoglycaemia in the absence of intact GCGR signaling (163). Thus, ghrelin may be a potential treatment for acute insulin-induced hypoglycaemia in type 1 diabetes patients.

## Insulin-like Peptide 5

Insulin-like peptide 5 (INSL-5) is predominantly expressed in the brain and colonic L cells (164, 165), with immunohistochemical staining and FACS analysis revealing that INSL-5 is overwhelmingly co-expressed with GLP-1 (164). Belonging to the Relaxin-peptide superfamily, INSL-5 has recently been identified as anorexigenic hormone. Secreted INSL-5 acts on the Relaxin/Insulin-like family peptide receptor 4 (RXFP4) (166), which is expressed along the GI tract, the nodose ganglion and the enteric nervous system (164), and inhibits adenylyl cyclase activity (167). Intraperitoneal, but not intracerebroventricular, administration of INSL-5 dose-dependently increases food intake in mice, indicating the peptide may exert its orexigenic effect by acting on peripheral targets, rather than via the CNS (164).

Strong evidence supports the role of INSL-5 as an energy sensor within the colon. Colonic *Insl5* and plasma INSL-5 levels are elevated during fasting in calorie-restricted mice and normalize upon refeeding (164). Increased colonic expression of *Isnl5* is also observed in germ-free (GF) mice, which lack a gut microbiome (168) and microbial-produced colonic short-chain fatty acids (SCFAs). As a consequence, GF mice have energy-depleted colonocytes due to the absence of their SCFA energy source, butyrate (169). Indeed, the introduction of a functional gut microbiome, which increases luminal SCFA availability, leads to reduced *Insl5* expression, in a manner similar to refeeding calorie-restricted mice (169). The role of INSL-5 as an energy sensor within the colon is not restricted to the availability of SCFAs, as *Insl5* expression in GF mice can also be reduced following HFD consumption, in which unabsorbed lipids provide an alternative energy source to colonocytes (168). As such, INSL-5 may serve as an important link between the gut microbiota and host in the context of metabolism.

The effect of INSL-5 on glucose homeostasis is less clear. While it was initially reported that mice deficient in *Insl5* were mildly glucose-intolerant (170), this appears to be age (170) and strain-dependent (164, 168). *Insl5*^−/−^ mice have impaired intraperitoneal glucose tolerance but superior insulin sensitivity and moderately reduced hepatic glucose production (168). The impact of INSL-5 on glucose control in mice also appears dependent on the mode of glucose delivery, as blood glucose or insulin levels were similar in *Insl5*^−/−^ mice compared to WT following an oral glucose test (164, 168). As oral but not intraperitoneal glucose administration stimulates the parasympathetic aspects of the gut-brain axis to centrally mediate hepatic glucose production (168), these findings suggest that INSL-5 may influence glucose homeostasis via direct actions on hepatocytes to influence hepatic gluconeogenesis. Studies on the insulinotropic action of INSL-5 have produced conflicting results (167, 171). As *Insl5* is not expressed in pancreatic islets (164, 168), any direct effects of endogenous INSL-5 on islets would appear to occur in an endocrine fashion. Circulating INSL-5 levels are estimated to be in the picomolar range (164, 172), which is several orders of magnitude lower than the EC_50_ of INSL-5 on RXRP4 (166) and the supraphysiological concentrations used in the majority of insulin secretion experiments may have contributed to the conflicting results.

## Concluding Remarks

**Figure 2:**
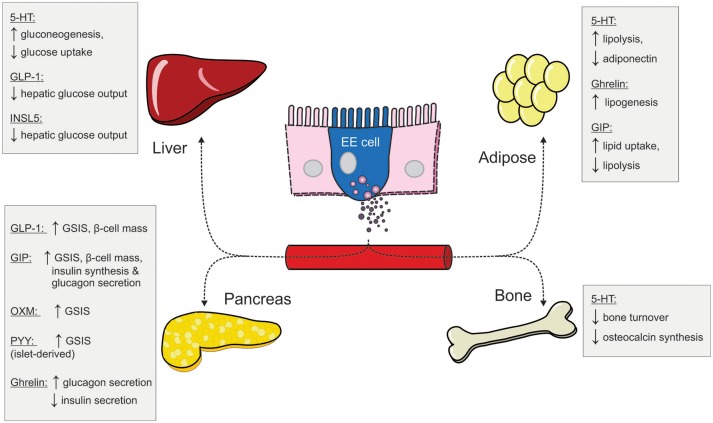
The peripheral metabolic effects of different gut hormones (5-HT, serotonin; EE, enteroendocrine; GIP, glucose-dependent insulinotropic hormone; GLP-1, glucagon-like peptide 1; INSL5, insulin-like peptide 5; OXM, oxyntomodulin; PYY, peptide YY; GSIS, glucose-stimulated insulin secretion).

Although enteroendocrine cells make up only 1% of the epithelial cell population along the GI tract (9), the hormones they secrete in response to one's nutritional status have profound impacts on peripheral metabolism (Figure 2). We have provided an overview of the metabolic actions of some of these gut hormones, including their role in maintaining glucose homeostasis and energy balance. Under fasting conditions, ghrelin and INSL5 levels are elevated to induce hunger and to prevent hypoglycaemia. Conversely, during the postprandial period, elevated GIP and GLP-1 levels augment postprandial insulin secretion to prevent hyperglycaemia. In addition to its insulinotropic effect, GLP-1 also act in concert with PYY and OXM to induce satiety (Figure 1). Moreover, some of the impressive metabolic gains from bariatric surgeries have been ascribed to alterations in the secretory profile of gut hormones. Altogether, the enteroendocrine system represents an attractive therapeutic target for treating metabolic disease as the pleiotropic effects of different gut hormones can be exploited individually.

## Author Contributions

ES and AM wrote the manuscript. DK and RY critically reviewed the manuscript. All authors approved the final version for publication.

### Conflict of interest statement

The authors declare that the research was conducted in the absence of any commercial or financial relationships that could be construed as a potential conflict of interest.
